# Retraction Note to: Differences in the expression profiles of claudin proteins in human nasopharyngeal carcinoma compared with non-neoplastic mucosa

**DOI:** 10.1186/s13000-018-0718-8

**Published:** 2018-06-25

**Authors:** Xiaowei Zhang, Haiming Wang, Qian Li, Yunpeng Liu, Peiqing Zhao, Tao Li

**Affiliations:** 1 Center for Translational Medicine; Department of Spinal Surgery, Central Hospital of Zibo, Affiliated with Shandong University, Gongqingtuan Road 54Hao, Zibo, Shandong Province China; 2 Department of General Surgery, People’s Hospital of Linzi District, Affiliated with Binzhou Medical College, Zibo, Shandong Province China; 3 Department of Spinal Surgery, Central Hospital of Zibo, Affiliated with Shandong University, Zibo, Shandong Province China; 4 Department of Thoracic Surgery, the First Hospital of Jilin University, Changchun, Jilin China

## Retraction note

The editor has retracted this article [1] because of flaws and inconsistencies in the methodology, reporting and interpretation of the data.

All authors agree to this retraction.
